# Impact of a mobile health intervention on health management among children with phenylketonuria based on a multi-theory model of the behavior change wheel theory and family health theory: protocol for a randomized controlled trial

**DOI:** 10.3389/fpsyt.2025.1583804

**Published:** 2025-05-27

**Authors:** Jie Wang, Sihan Yang, Jing Wang, Bo Zhu, Yan Wang, Lirong Zhao, Wenli Wang, Ge Li, Fei Wang, Xiaohua Wang

**Affiliations:** ^1^ Department of Genetics, Inner Mongolia Maternity and Child Health Care Hospital, Hohhot, Inner Mongolia, China; ^2^ School of Public Health, Peking University, Beijing, China; ^3^ School of Health, Shandong University of Traditional Chinese Medicine, Jinan, Shandong, China; ^4^ Shandong Key Laboratory of Reproductive Research and Birth Defect Prevention, Gynecology Department, Shandong Provincial Hospital Affiliated to Shandong First Medical University, Jinan, Shandong, China; ^5^ Gynecology Department, Inner Mongolia Maternity and Child Health Care Hospital, Hohhot, Inner Mongolia Autonomous Region, China; ^6^ Pediatric Health Care Department, Inner Mongolia Maternity and Child Health Care Hospital, Hohhot, Inner Mongolia, China

**Keywords:** phenylketonuria, mobile health (mHealth), behavior change wheel, family health theory, health management

## Abstract

**Background:**

Phenylketonuria (PKU) is a hereditary metabolic disorder caused by mutations in the phenylalanine hydroxylase (*PAH*) gene, leading to the accumulation of phenylalanine (Phe) in the blood. Without timely treatment, PKU patients may develop irreversible intellectual and neurocognitive deficits. The primary treatment for PKU is a strict low-Phe diet, which must be initiated in early life to achieve better outcomes. Therefore, parents and parents’ caregivers play a crucial role in dietary management. However, poor dietary adherence and inadequate health knowledge significantly affect treatment efficacy. Mobile health (mHealth) interventions, as an emerging health management approach, offer personalized, low-cost, and highly accessible solutions. This study aims to develop and evaluate an mHealth intervention model based on the Behavior Change Wheel (BCW) theory and family health theory to improve health management for children with PKU.

**Methods:**

This single-center, single-blind randomized controlled trial will be conducted at the Inner Mongolia Maternity and Child Health Care Hospital in China from March to September 2025. Participants will include children with PKU and their parents’ caregivers, who will be randomly assigned to either the intervention group (receiving mHealth interventions via WeChat) or the control group (receiving routine health education). The intervention will include health education, dietary management, regular monitoring reminders, and online consultation services. The primary outcome will be the serum phenylalanine and tyrosine levels of the children. Secondary outcomes will include nutritional indicators, intellectual development indicators, and the caregivers’ health knowledge, compliance, and mental health. Data collection will occur at baseline and at 3 and 6 months post-intervention.

**Discussion:**

By integrating the BCW and family health theories, this study innovatively develops an mHealth-based health management intervention model for PKU. This model is expected to enhance family health management capabilities and improve clinical outcomes and quality of life for children with PKU. If effective, this model can be extended to other regions to provide more convenient and efficient health management solutions for PKU patients.

## 1 Introduction

Phenylketonuria (PKU) is an inherited metabolic disorder caused by pathogenic mutations in the phenylalanine hydroxylase (*PAH*) gene, leading to decreased *PAH* activity and increased blood levels of phenylalanine (Phe) in the blood. Based on blood Phe concentrations, PKU can be categorized as moderate hyperphenylalaninemia (120-360 μmol/L), mild PKU (360-1200 μmol/L), or classical PKU (≥ 1200 μmol/L) according to the Rare Disease Diagnosis and Treatment Guidelines (2019).

In PKU patients, Phe builds up in the blood, cerebrospinal fluid, and brain, because it cannot be converted into tyrosine (Tyr). This can lead to varied degrees of irreversible intellectual and neurocognitive deficits if therapy is delayed (1). Globally, approximately 450,000 individuals are affected by PKU, with an average newborn prevalence of roughly 1 in 23,930 (2). Since 2009, PKU has been a required screening item in China’s Neonatal Disease Screening Management Measures. Affected infants’ prognosis can be greatly improved by early diagnosis by neonatal screening and subsequent low-Phe dietary intervention, which plays an essential role in lowering birth abnormalities and enhancing population health.

The main treatment for PKU is strict, long-term low-phenylalanine dietary therapy (3). When blood Phe levels are over 360 μmol/L, patients are advised to start a low-phenylalanine diet. Blood Phe levels should be regularly monitored to keep them between 120 and 360 μmol/L. Effective low-phenylalanine dietary management is, however, severely hampered by variables such poor dietary adherence, limited access to information on Phe content in foods, and low health literacy among patients and their parents, according to existing studies (4). Furthermore, the diet’s restriction raises the possibility of shortages in vital nutrients including vitamin B12, vitamin D, and folate, requiring frequent supplementation (5–7), which makes low-phenylalanine diet even more difficult.

The crucial role of parents and main caregivers is shown by the fact that early commencement of low-phenylalanine dietary therapy is essential for improved outcomes in PKU children. Few studies have examined the effects of interventions aimed at parents or main caregivers on health management among children with PKU, despite studies demonstrating that offering parents nutritional advice and skill training can improve the management of children who need special diets (8, 9). The blood Phe levels of PKU infants appear to be inversely correlated with parental understanding of PKU and dietary control, according to cross-sectional studies (10–12). Additionally, two intervention trials that focused on the main caregivers of children with PKU (4, 13) showed that health education combined with low-Phe dietary management is more successful than dietary intervention alone at lowering blood Phe levels.

However, traditional intervention methods for parents including in-person training sessions and written materials have limited scalability because of budget, time, and human resource limitations (14–17). Mobile health (mHealth), which have been extensively used in mental health, chronic illness management, and other fields, are becoming more and more important in modern health management, which provides more individualized, affordable, and accessible interventions (18). With the characteristics of easy to use, no need to additional download, and strong interactivity, WeChat can improve patients’ health outcomes, quality of life, and treatment compliance, while offering the possibility of individualized health management (19–23).

**Figure 1:**
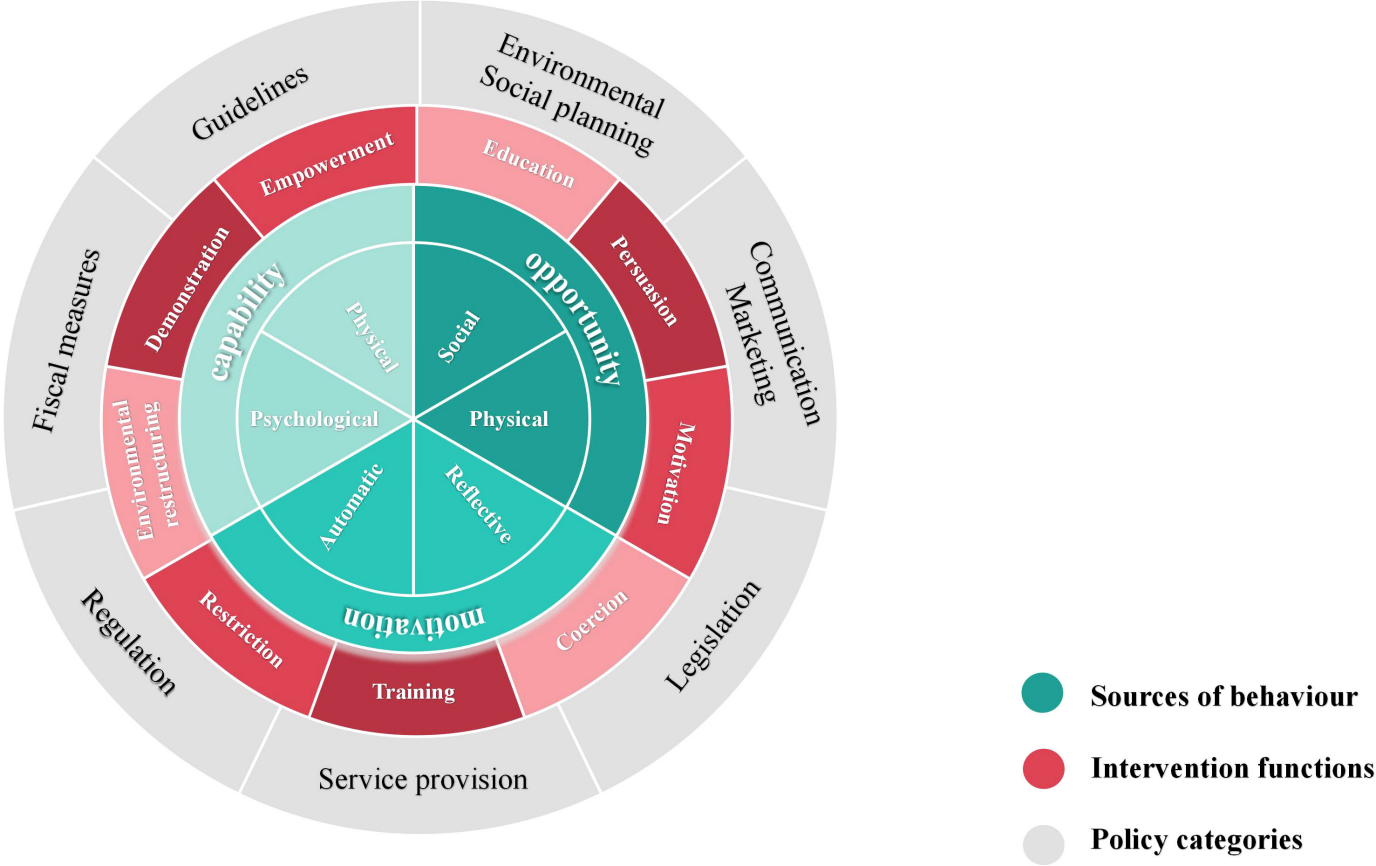
Schematic diagram of the Behavior Change Wheel (BCW) theory.

Susan Michie et al. developed the Behavior Change Wheel (BCW) theory in 2011 (24), which combines 19 behavior change theories to assist intervention planners in identifying obstacles to behavior change and creating the most effective comprehensive intervention plan. The BCW framework consists of three layers, as shown in **Figure 1**. The inner layer offers a thorough examination of the obstacles to changing one’s own behavior by examining the interaction of capability, opportunity, and motivation in shaping behavior (COM-B model). Nine intervention functions, education, persuasion, motivation, coercion, training, restriction, environmental restructuring, demonstration and empowerment, are identified by the intermediate layer to help direct the design of interventions. Seven categories for macro policy interventions are included in the outer layer to support the execution of intervention functions.

Additionally, Susan Michie et al. proposed a three-stage and eight-step procedure for putting the BCW theory into practice, which includes comprehending the behavior, choosing possible interventions, and establishing the content of the interventions to carry them out. The BCW theory has been widely applied in chronic disease management, dietary and nutritional management, health screening, medication adherence, and other health promotion studies (25–28). Several studies have successfully designed mHealth interventions using the BCW theory, showing positive outcomes (19, 29, 30).

Given that for children with PKU, parents are the primary factors influencing diet compliance and blood Phe control, this study combines the BCW theory with family health theory, targeting parents of PKU children as the main intervention subjects. Sharon Denham first innovatively proposed the concept of “family health” in 2003 (31). Weiss-Laxer et al. expanded on it by using the Delphi method to create a more thorough and methodical concept framework for “family health,” characterizing it as “A resource at the level of the family unit that develops from the intersection of the health of each family member, their interactions and capacities, as well as the family’s physical, social, emotional, economic, and medical resources” (32).

**Figure 2:**
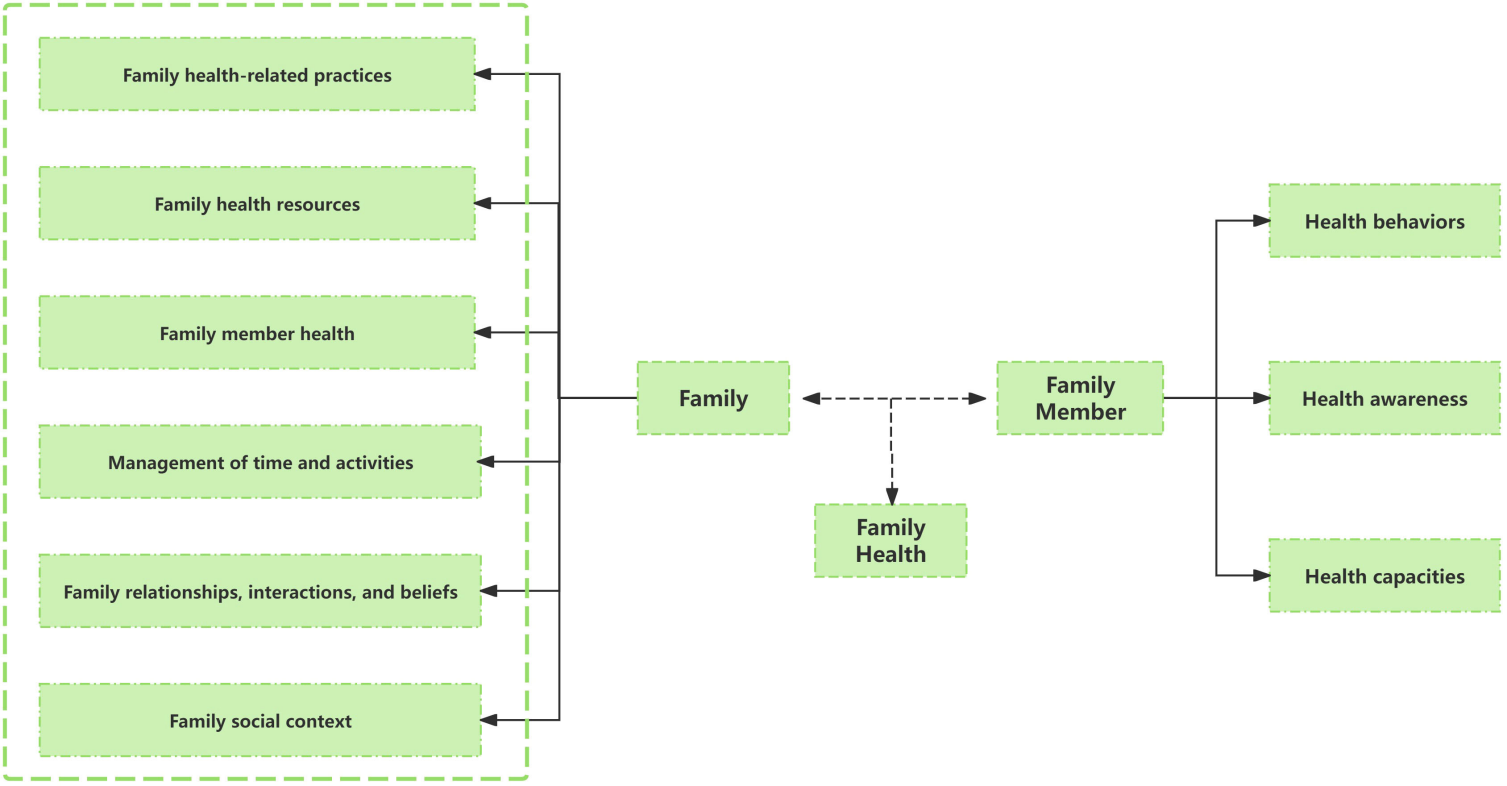
Schematic diagram of the family health concept framework.

The family health concept framework was thoroughly examined in this study, as shown in **Figure 2**. Notably, family health is not simply the sum of individual health but encompasses six domains: Family relationships, interactions, and beliefs; Family social context; Family member health; Family health-related practices; Family health resources; Management of time and activities. These domains include both the sharing of internal family resources and the acquisition of external resources. These internal and external elements impact each family member’s health behaviors, awareness, and capacities through interactions, which in turn impacts the family’s overall health. Evidence suggest that family-centered health promotion programs play a significant role in primary, secondary, and tertiary prevention, and are effective and cost-effective in public health practice (33, 34).

In conclusion, given that mHealth can effectively break through the time and space limitations and provide personalized intervention programs, this study aimed to develop a mHealth intervention model based on BCW and family health theory, which involved multiple intervention strategies such as improve the parents’ knowledge of PKU, follow a low-Phe die, regular monitor of blood and so on. It is hoped to provide a clinical basis for mHealth intervention to improve health management among children with phenylketonuria PKU.

Specific objectives are:

1. To evaluate the impact of the mHealth based on BCW and family health on clinical outcomes in children with phenylketonuria.
2. To determine the effectiveness of the intervention program in improving health behavior management of children with phenylketonuria.

## 2 Methods

### 2.1 Trial design and setting

The research will be conducted at the Inner Mongolia Maternity and Child Health Care Hospital in China from March 2025 to September 2025, employing clinical reagent testing and internationally recognized scales for quantitative investigation.

The study population consists of families of children with PKU, who will be divided into two groups: Group A will be the mHealth intervention group, while group B was the control group. The intervention group will receive mobile health interventions through WeChat group chats. The control group, on the other hand, will simply get regular health education, which includes an oral presentation on PKU by a professional physician at the time of enrollment, educating the parents of PKU children on how to manage their diets low in phenylalanine. A printed handbook on PKU disease and daily management knowledge will also be given to them, but they won’t have access to online education and training, frequent reminders for monitoring, or online consultation services.

**Figure 3:**
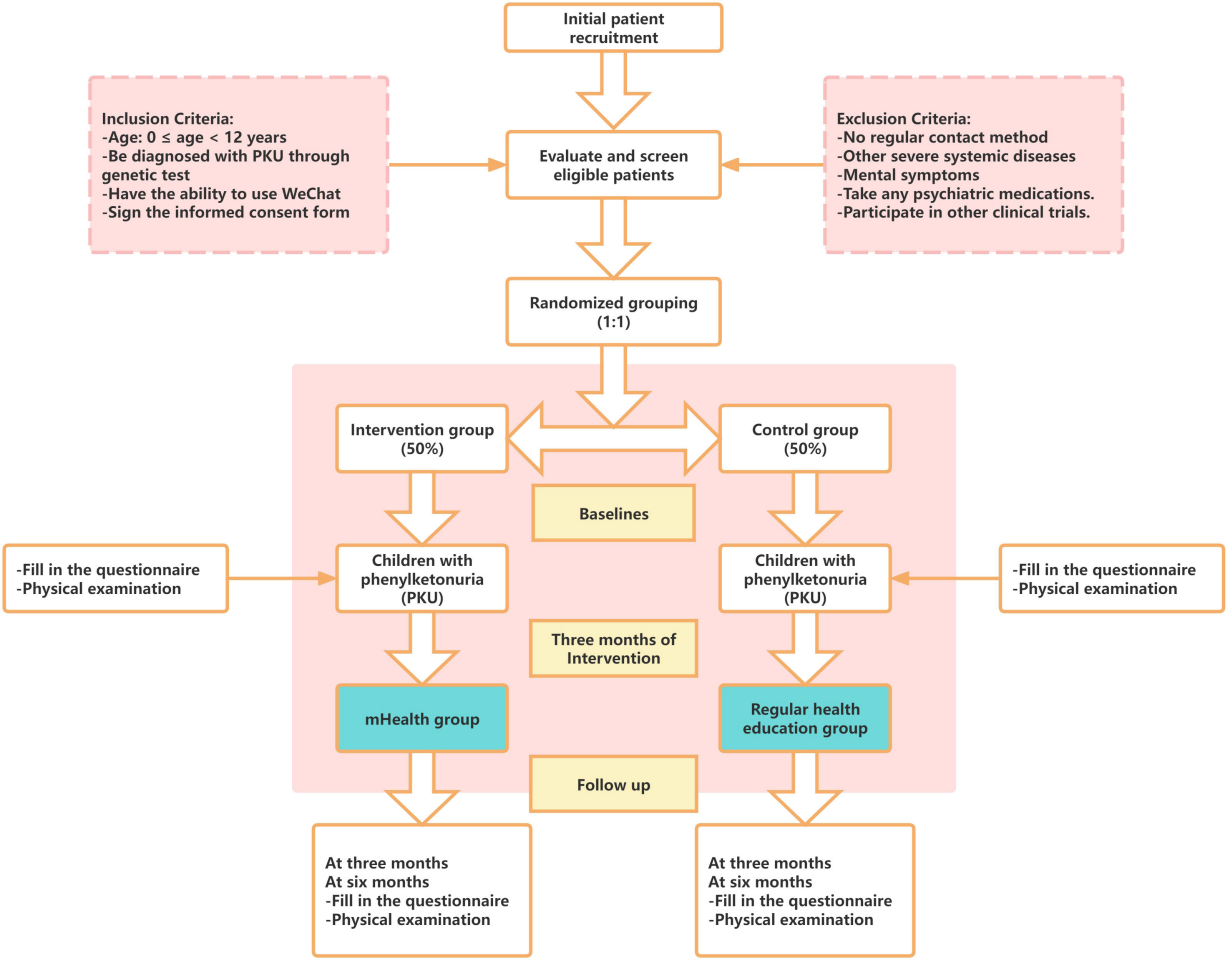
Flow chart of patient recruitment and study implementation.

The intervention will last for 6 months, with clinical indicators, intelligence tests, and questionnaire surveys being conducted at baseline, as well as at 3 and 6 months after the start of the intervention to examine the effectiveness of the intervention on the health management of children with PKU. The flow chart of the study is shown in **Figure 3** and **Table 1**.

**Table 1:** Timeline for study enrollment, intervention, and evaluation.

Study Period	Recruitment	Intervention		Follow-up
Timepoint	-T1	0M	3M	6M
ENROLMENT: eligibility screen	✓			
Informed consent	✓			
ASSESSMENTS: sociodemographic variables		✓		
Anthropometric variables		✓	✓	✓
Intellectual development variables		✓	✓	✓
PKU knowledge		✓	✓	✓
Parents’ compliance with health management		✓	✓	✓
Parents’ mental health variables		✓	✓	✓
Family environment variables		✓	✓	✓

### 2.2 Study participants

The participants of this study are all PKU children and their parents’ caregivers, such as parents, who meet the inclusion criteria and have been screened by the Inner Mongolia Maternal and Child Health Care Hospital in China by the end of February 2025. Each participant will begin the formal intervention after being informed face-to-face by a hospital doctor and signing the research informed consent form. This study has been approved by the Ethics Committee of Inner Mongolia Maternity and Child Health Care Hospital, Hohhot, Inner Mongolia Province.

Inclusion Criteria:

1. Age: 0 ≤ age < 12 years, regardless of gender.
2. The child must meet the diagnostic criteria for PKU as outlined in the Rare Disease Diagnosis and Treatment Guidelines (2019) and be confirmed as a PKU patient through genetic testing.
3. The parents’ caregivers, such as the child’s parents, must have the ability to use WeChat and possess normal language communication skills.
4. They must understand and agree to participate in the intervention, be willing to fully cooperate with the intervention measures and follow-up examinations, and sign the informed consent form.

Exclusion Criteria:

1. The parents’ caregivers do not have a regular contact method or are unable to use WeChat daily.
2. The child or parents’ caregivers have other severe systemic diseases or significant physical functional impairments.
3. The child or parents’ caregivers exhibit mental symptoms such as unclear consciousness, language expression difficulties, or uncooperativeness.
4. The child or parents’ caregivers are taking any psychiatric medications.
5. The child or parents’ caregivers are already participating in other clinical trials.
6. Any other reasons deemed unsuitable for participation in the trial.

### 2.3 Sample size

The sample size was calculated based on the primary metabolic indicator, serum Phe concentration. This study is a randomized controlled trial, with the intervention group receiving mobile health interventions and the control group receiving regular health education. The sample size ratio between the two groups is 1:1. According to a randomized controlled trial on PKU children based on a mobile health app (35), the blood Phe concentration in the control group was 360 μmol/L, while that in the intervention group was 260 μmol/L, with a standard deviation of 100 μmol/L in both groups. Using a two-tailed test with α = 0.05 and β = 0.1, the power (1-β) was set at 90%. Calculations performed using PASS 15 indicated that a minimum of 23 participants per group was required. Accounting for a 10% attrition rate, each group was expanded to 25 participants, resulting in a total minimum sample size of 50 participants.

### 2.4 Randomization, allocation, and blinding

This research project is a single-blind, single-center randomized controlled trial to be conducted at Inner Mongolia Maternity and Child Health Care Hospital, Inner Mongolia, China. A simple random sampling method is employed for children diagnosed with PKU at our hospital. Excel software is used to create random number sequences. Participants are divided into two groups (the experimental group and the control group) using the envelope approach, which is based on the sorted order of these numbers. Randomly numbered pieces of paper are put into sealed envelopes. After being enrolled, participants open their own envelopes and discover their group assignment after receiving them in the correct order based on their given numbers. A committed staff member supervises and records the envelope distribution and opening, and safely preserves unused envelopes to maintain the integrity of allocation concealment and the single-blind design of the study.

### 2.5 A Multi-Theory Model based on BCW and family health

**Figure 4:**
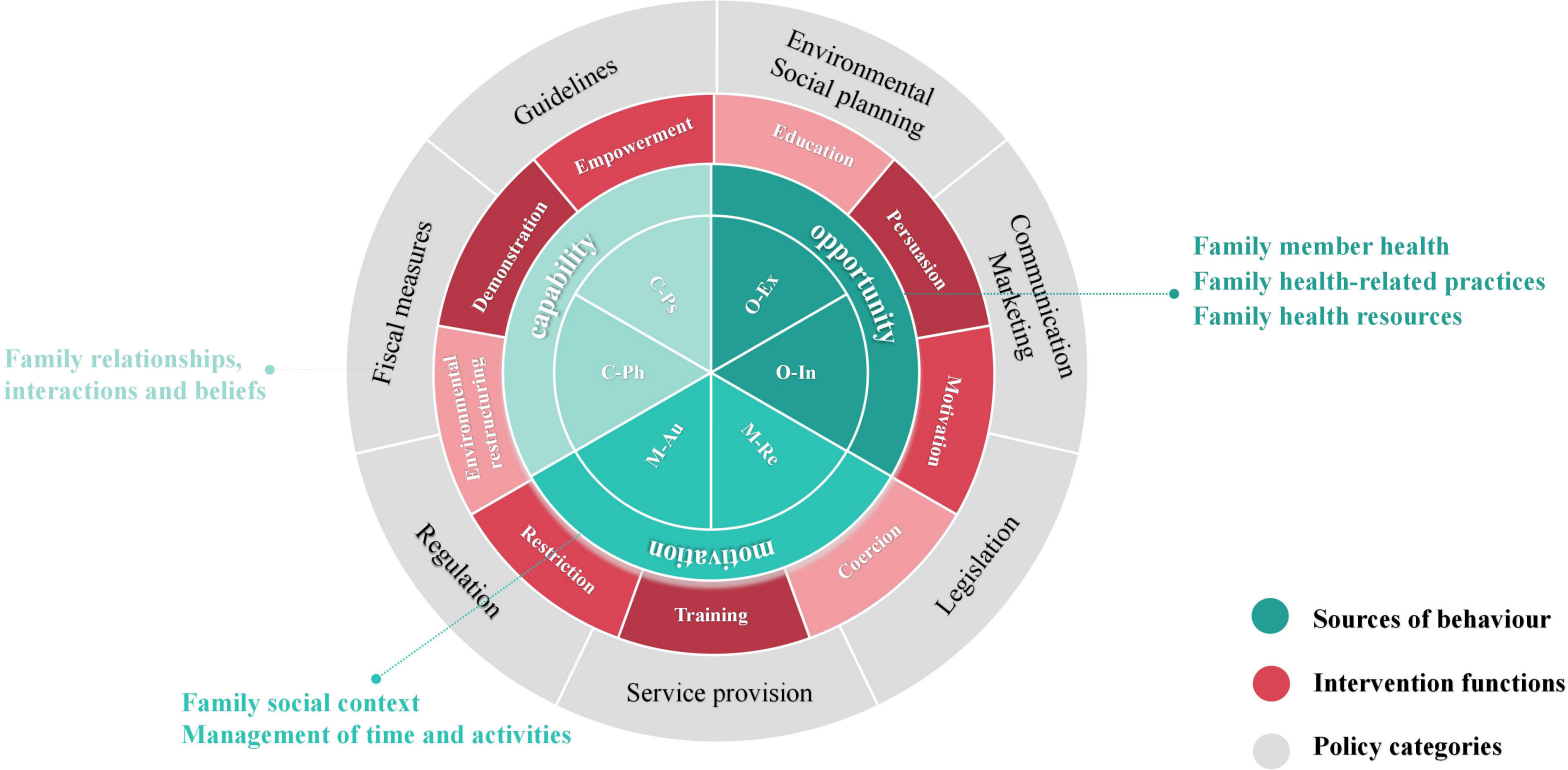
Schematic diagram of a multi-theoretical model.

Because family has an overarching role in individual wellbeing, and the strength of communities and society (36), the combination of the BCW and the Family Health theory provides a novel perspective and pathway for health behavior interventions. By applying the BCW theory from the individual level to the six domains of the Family Health, a Multi-Theory Model (shown in **Figure 4**) is formed, which can offer more effective intervention strategies for improving the health behaviors of individual family members as well as the family as a whole. The three levels of the BCW theory will be used to discuss how these two theories can be combined and used below.

#### 2.5.1 Expansion of the COM-B model in family health: the COM-FB model

The inner layer of the BCW theory is the COM-B model, which explores three components of behavior (B): capability (C), opportunity (O), and motivation (M). These components can be mapped to the six domains of the Family Health Theory framework, thereby extending the model to the family level and forming the COM-FB model. Specifically:

Capability (C): Family member health, Family health-related practices, and Family health resources of the Family Health theory specify requirements for capability. This includes physical capability (C-Ph), such as caring for patients, providing appropriate food, and regular monitoring, as well as psychological capability (C-Ps), such as disease knowledge and understanding.

Opportunity (O): Opportunity refers to environmental factors that facilitate behavior change, including social opportunities such as interpersonal relationships and physical opportunities such as time and resources. Family social context, Management of time and activities of the Family Health theory defines opportunities at the family level. This includes external opportunity (O-Ex), such as family safety and home environment, and internal opportunity (O-In), such as time allocation for work and family activities.

Motivation (M): Family relationships, interactions and beliefs of the Family Health theory addresses motivation at the family level. This includes automatic motivation (M-Au), such as family cohesion and a sense of belonging, and reflective motivation (M-Re), such as beliefs and values regarding caregiving.

#### 2.5.2 Application of the nine intervention functions in family health

According to the BCW theory, once the family-level COM-FB model is established, specific intervention strategies can be designed based on the nine intervention functions: education, persuasion, motivation, coercion, training, restriction, environmental restructuring, demonstration, and empowerment. For example, teaching parents to improve disease awareness and understanding, training to improve the ability to take a low-Phe diet, and setting incentive mechanism to improve the enthusiasm of regular monitoring.

#### 2.5.3 Application of external resources and policy support in family health

The outer layer of the BCW theory consists of seven policy categories, emphasizing the supportive role of external resources in enabling intervention functions. Therefore, after selecting the nine intervention functions, external resources can be utilized to facilitate their implementation. For example, society can distribute printed or electronic materials for education, relevant institutions can develop disease guidelines, governments can provide financial support such as medical insurance, and primary healthcare institution can offer more affordable and high-quality consultation services, making it easier for family to access health education resources.

### 2.6 Development of mHealth intervention based on the Multi-Theory Model

The Multi-Theory Model, which was created by combining the BCW theory and the Family Health theory, will serve as the foundation for the study. Using the WeChat, a mobile health intervention program will be developed for families of PKU children (including the children and their parents’ caregivers, such as parents) to explore its effectiveness in the health management of PKU children, including metabolism, nutrition, and intellectual development. According to the specific stages of applying the BCW theory, the intervention strategies for PKU families are as follows:

#### 2.6.1 Stage 1: examine the obstacles to changing behavior of PKU families based on the COM-FB model

C-Ps (Psychological Capability): Refers to the cognitive level of PKU children and their parents’ caregivers regarding phenylketonuria, including knowledge of the disease, specific requirements of a low-Phe diet, and the importance of regular monitoring. Currently, some parents of PKU children lack sufficient awareness of the importance of PKU health management, which is one of the main reasons for poor dietary and follow-up compliance.

C-Ph (Physical Capability): Refers to the skill level of PKU children and their parents’ caregivers in preparing and maintaining a low-Phe diet. At present, some parents’ caregivers lack professional training and are deficient in skills such as food pairing for a low-Phe diet, understanding ideal Phe intake, and supplementing other nutrients in daily feeding.

O-Ex (External Opportunity): Refers to the access of PKU families to external medical resources, including nearby medical institutions that conduct newborn PKU screening and regular blood Phe monitoring, professional physicians providing consultation services, and educational materials on PKU knowledge and skills training. Additionally, it includes the convenience of accessing information on Phe content in foods.

O-In (Internal Opportunity): Refers to the time and energy allocated by family members for the health management of PKU children, including the time distribution of parents’ caregivers and family communication. This is related to the level of importance family members place on the child and their compliance with health management. Currently, some parents have limited energy for the health management of their children, with insufficient attention paid to diet, follow-up, and growth and development monitoring, significantly affecting the effectiveness of PKU health management.

M-Au (Automatic Motivation): Refers to the cohesion, sense of belonging, and responsibility among family members, which serve as important internal driving forces for strict and continuous PKU health management.

M-Re (Reflective Motivation): Refers to the family’s trust in the effectiveness of health management for PKU children. Currently, some families hold pessimistic attitudes toward the intellectual development and quality of life of PKU children and lack confidence in the effectiveness of health management measures such as a low-Phe diet, greatly reducing their motivation for daily management.

#### 2.6.2 Stage 2: select intervention functions

Based on the analysis of the main barriers to improving the behavioral impact on families of children with PKU in Stage 1, interventions are selected from the nine major intervention functions, combined with seven policy categories to facilitate their implementation.

Education: Provide printed or electronic PKU educational materials.

Persuasion: Professional physicians educate the parents’ caregivers of children with PKU based on PKU guidelines; Set up timed reminders for blood Phe monitoring.

Motivation: Provide feedback on daily dietary habits and regular monitoring progress; establish family reward mechanisms such as points, prizes, and honorary titles.

Training: Professional physicians train the parents’ caregivers of children with PKU on low-Phe dietary strategies based on PKU guidelines, including questions such as what to eat, how much to eat, and how to eat.

Environmental Restructuring: Improve accessibility to medical resources and information on food Phe content, including setting up convenient channels for consulting professional physicians, providing Phe content tables for common foods, and automated Phe content calculation tools.

Demonstration: Regularly organize family exchange meetings for PKU patients to share health management experiences.

Empowerment: Develop low-Phe dietary plans according to PKU guidelines; Conduct regular follow-up monitoring of blood Phe levels, nutrition, intellectual development, and quality of life in local medical institutions.

#### 2.6.3 Stage 3: determine intervention content and implementation options—mobile health intervention based on the WeChat

Based on the analysis and selections from the previous two stages, and after comprehensively considering affordability, practicability, effectiveness acceptability, safety, and equity, the final decision is to utilize WeChat group chats for remote mobile health intervention. The group chat will incorporate the following four functions to achieve the seven interventions outlined in Stage 2:

1. *Health education module and real-time reminder function*. Through the WeChat group, educational materials in various formats (e.g., illustrations, videos) will be distributed weekly to provide PKU knowledge and health education. Topics include basic disease knowledge, the importance of health management, low-Phe diets, and regular follow-up monitoring. Personalized reminder notifications will also be set up to encourage timely completion of dietary records and regular medical check-ups.
2. *Diet uploading and phenylalanine content query function*. Parents will input the types and quantities of food consumed by their child each day into the WeChat group chat. The system will automatically calculate the daily Phe intake, notify parents if the intake exceeds the recommended limit, and provide adjustment suggestions. Additionally, the WeChat group will include a built-in food Phe database containing Phe content for common foods across three meals, along with recommended low-Phe meal combinations.
3. *Regular feedback and incentive mechanism*. The group chat will record the dietary logs and compliance status uploaded by parents. At the end of each month, virtual rewards such as badges and titles will be issued based on the monthly records to motivate parents to continue maintaining healthy management behaviors.
4. *Family interaction and online consultation function*. Parents can interact with other PKU families in the group chat to share health management experiences. Additionally, the group chat will offer an online consultation feature, allowing parents to seek advice from professional physicians in the group at any time.

### 2.7 Health education materials

#### 2.7.1 Sources of health education materials

The educational materials used for health education come from the Internet, including videos and illustrations. The illustrations are obtained from WeChat, Baidu Baike, Wikipedia, doctor’s Q&A, or domestic and international databases such as CNKI. The videos are primarily animations and are sourced from platforms such as WeChat and Baidu Baike. Case information is derived from Baidu documents or literature. All educational materials are required to be scientifically sound and supported by relevant references.

#### 2.7.2 Screening of health education materials

All health education materials are examined and assessed by experts in the field of PKU, and screened based on expert feedback. Selected materials are compiled into a health education database. The database includes the article title, relevant links, source, and material type. To make it easier to create individualized treatment regimens for patients, these resources were divided into four major parts according to their principal content: disease knowledge, low-Phe diet, routine monitoring, and mental health.

### 2.8 Outcomes

Primary Outcomes:serum phenylalanine and tyrosine levels in children with PKU.

Secondary Outcomes:

1. Nutritional indicators: Height/length (cm), weight (kg), head circumference (cm), the concentration of hemoglobin, prealbumin, albumin, complete blood count (red blood cells, white blood cells, platelets), serum iron, ferritin, and 25-Hydroxy vitamin D3.
2. Intellectual development indicators: For children aged ≤6 years, professional physicians will use the 0–6 Years Child Developmental Behavior Assessment Scale for evaluation. For children aged >6 years, IQ will be assessed using the Chinese version of the Wechsler Intelligence Scale for Children, Fourth Edition (WISC-IV).

In addition, considering that the health management of children with PKU may impose psychological and quality-of-life burdens on their parents’ caregivers (37, 38), the study also focuses on the following indicators for parents’ caregivers: PKU disease knowledge, compliance, mental health, family health, and family atmosphere, etc. Specific details are as follows:

1. Changes in PKU knowledge.
2. Changes in parental compliance with health management: Measured by the number of dietary Phe concentration logs and the number of blood tests during the intervention period (39).
3. Impact of health management on parental mental health: Includes levels of anxiety and depression, self-efficacy, and parenting stress.
4. Changes in the family environment of PKU children: Includes specific behavioral changes such as family communication and broader indicators such as family health.

Socio-demographic variables will be collected from patients at baseline. Clinical reagent testing and questionnaires will be conducted at baseline as well as at 3 and 6 months of follow-up, with data collection times shown in **Table 2**.

**Table 2:** Data collection components and collection timeline.

Type of research	Data collection		Timepoint		
			0M	3M	6M
Quantitative research	Sociodemographic variables	Gender, age, parental education level, parental occupation, monthly household income per capita, only-child status, presence of other PKU patients in the family	✓		
	Anthropometric variables	Serum phenylalanine(Phe)and tyrosine(Tyr)levels, height/length (cm), weight (kg), head circumference (cm), the concentration of hemoglobin, prealbumin, albumin, complete blood count, serum iron, ferritin, and 25-Hydroxy vitamin D3	✓	✓	✓
	Intellectual development variables	For children aged ≤6 years, professional physicians will use the 0–6 Years Child Developmental Behavior Assessment Scale for evaluation. For children aged >6 years, IQ will be assessed using the Chinese version of the Wechsler Intelligence Scale for Children, Fourth Edition (WISC-IV)	✓	✓	✓
	PKU knowledge	Questionnaire: Changes will be measured by a self-designed PKU theory scale	✓	✓	✓
	Parents compliance with health management	Measured by the number of dietary Phe concentration logs and the number of blood tests during the intervention period	✓	✓	✓
	The level of anxiety and depression on parents	Questionnaire: A simplified version of the Anxiety Depression Scale (PHQ-4)	✓	✓	✓
	The level of self-efficacy on parents	Questionnaire: A simplified version of New General Self-Efficacy Scale (NGSES-SF)	✓	✓	✓
	The level of parenting stress on parents	Questionnaire: The Parenting Stress Index (PSI-SF-15) and the Guilt about Parenting Scale (GAPS)	✓	✓	✓
	Family communication	Questionnaire: A Short Form of the Family Communication Scale (FCS-SF)	✓	✓	✓
	Family health	Questionnaire: A Short Form of the Family Health Scale (FHS-SF)	✓	✓	✓

### 2.9 Questionnaires

Socio-demographic and clinical data will be collected using self-designed questionnaires and the medical record system. Socio-demographic variables included gender, age, parental education level, parental occupation, monthly household income per capita, only-child status, and presence of other PKU patients in the family. Clinical variables included age at diagnosis, historical serum Phe concentrations, and intellectual development level.

Changes in PKU knowledge will be assessed using a self-designed PKU theory scale. This scale is adapted from the study conducted by Öztürk F et al. on the PKU knowledge of parents (40). It comprises 10 questions related to PKU, all of which are based on the information provided in Nelson’s textbook Classical Pediatrics to assess both correct and incorrect knowledge. Each question is scored as 1 point, with the total score ranging from 0 to 10. The development of the scale will be undertaken by the researcher, who will also complete its final validation. To determine the appearance validity and content validity, a pre-test will be administered to 20 randomly selected participants who did not take part in the final trial. These participants will be chosen from the recruited pool. The scale will be refined through pre-testing and expert review to ensure its robustness and reliability.

Depression and anxiety will be assessed using an ultra-brief screening scale for anxiety and depression (PHQ-4), which is a short, self-report scale used to assess an individual’s symptoms of depression and anxiety over the past 2 weeks (41). The scale comprises two dimensions: depression and anxiety, with a total of four items. The first two items assess depressed mood, while the last two evaluate anxiety. Each item is rated on a 4-point scale, with “not at all”, “a few days”, “more than half the days” and “almost every day,” corresponding to a score of 0, 1, 2, and 3, respectively, and the total score ranges from 0 to 12. Different levels of psychological problem were defined with scores: normal (0~2), mild (3~5), moderate (6~8), and severe (9~12). The Cronbach’s alpha of the PHQ-4 was 0.865, which is of high validity and reliability, in a study conducted in Chinese parents (42). Due to its brevity and efficiency, the PHQ-4 is a practical tool for healthcare professionals to quickly and effectively assess anxiety and depression in target populations.

Self-efficacy will be assessed by New General Self-Efficacy Scale-Short Form (NGSES-SF). The NGSES was first developed by Chen, G. to measure individuals’ perception of their ability to perform across a variety of different situations in 2001. Wang Fei et al. later adapted the short form (NGSES-SF) and validated its reliability and validity in a Chinese population (43). The NGSES-SF is a 3-item, 5-point Likert-type scale, with responses ranging from 1 (very disagree) to 5 (very agree). Higher scores indicate a greater level of self-efficacy.

Parenting stress will be measured by the Parenting Stress Index (PSI-SF-15) and the Guilt about Parenting Scale (GAPS).The PSI was first developed by Abidin to measure parenting stress perceived by the caregivers in 1995 (44). Jie Luo et al. (45) later adapted the short form (PSI-SF-15) and validated its reliability and validity in Chinese parents. The PSI-SF-15 comprises three dimensions: parental distress (PD), parent-child dysfunctional interaction (PCDI), and difficult child (DC). It is a 5-point Likert-type scale with 15 items, ranging from 1 (very disagree) to 5 (very agree), with higher scores indicating a higher degree of parenting stress perceived by caregivers. The Guilt about Parenting Scale (GAPS) was developed by Haslam et al. in 2019, to measure the degree of guilt caused by conflicts between parents’ work and family responsibilities (46). It consists of 10 items, scored from 1 (completely disagree) to 7 (completely agree), all with positive scoring, and higher scores indicate a higher level of parental parenting guilt. After verification, the Cronbach’s α of this scale was 0.89, indicating good reliability and validity.

Family health will be measured by the A Short Form of the Family Health Scale (FHS-SF), which mainly includes four dimensions of family/social and emotional health processes, family healthy lifestyles, family health resources, and social support outside the family. It is used to comprehensively assess the family health status. To reduce subjects’ fatigue responses and increase the completion rate in practical studies, Crandall et al. further developed the short form of the Family Health Scale (47). The scale consists of ten items, and the response options are usually on a five-point scale ranging from “strongly disagree” to “strongly agree”. Among them, the three items of the family health resource dimension are reverse-scored. Fei Wang et al. adapted the Chinese version of the FHS-SF based on this, with Cronbach’s α of 0.83, which is of high validity and reliability, in a study involving the Chinese population (48).

Family communication will be assessed by A Short Form of the Family Communication Scale (FCS-SF), which has four items measuring a single dimension: family communication level. It is a 4-point Likert scale, with responses ranging from 1 (strongly disagree) to 4 (strongly agree).The Family Communication Scale has demonstrated good internal consistency and reliability in the localization process in Greece (49), Spain (50) and China (51). To facilitate quicker and more convenient use in research and clinical practice, Siyuan Fan et al. developed the FCS-SF based on the FCS-10. The FCS-SF retains the psychometric advantages of the FCS-10 while being simplified to assess the overall level of family communication more efficiently. It has shown good psychometric properties in Chinese populations, with a Cronbach’s α of 0.917.

## 3 Statistical analysis

Statistical analysis will be performed using SPSS Statistics 26.0. Categorical data will be expressed as counts and percentages (n, %). Continuous data will be presented as mean ± standard deviation (x¯ ± s). The Mann-Whitney U test will be used to compare changes in each indicator between the two groups at 3 months and 6 months post-intervention relative to baseline. The Wilcoxon test will be employed to compare differences between the two groups at baseline and across four follow-up time points. Baseline demographic characteristics between the two groups will be compared using the chi-square test. A two-tailed test will be applied, and a P-value < 0.05 will be considered statistically significant.

If a reasonable amount of missing data is present, the data summary will indicate that the data are missing at random. In such cases, all analyses will incorporate multiple imputation procedures using baseline variables as auxiliary variables to address missing data. Linear regression and logistic regression will be selected for the imputation process, and the final analysis results will be combined to complete the imputation and analysis procedures.

### 3.1 Data collection

In this study, all data were gathered by professionally trained physicians from the Inner Mongolia Maternity and Child Health Care Hospital. These physicians conducted assessments of metabolic, nutritional, and intellectual development levels in PKU patients in strict adherence to the protocol guidelines. They also provided on-site assistance to families to help them complete the questionnaires. Furthermore, each family of PKU patients was assigned a unique code and registered to ensure that data from all follow-up points were accurately recorded on the pre-designed subject registration forms. The forms included fields for date and signature at the end to facilitate review and retrospective verification. Lastly, during the data analysis phase, a second review was conducted to identify and exclude any illogical or incomplete data entries.

### 3.2 Data management

The researchers stored all trial-related information, including subject identification codes, source data, and investigator files, along with associated communications, in a dedicated database for the study. Upon completion of the trial, the source data within the database and all pertinent study documents will be preserved in compliance with legal and regulatory requirements.

### 3.3 Safety

All adverse events (SAEs) or adverse events (AEs) reported by patients or detected by investigators will be collected during the trial period and must be recorded on the corresponding pages of the registry. All SAEs and AEs that occur after the patient signs the informed consent form will be recorded on the pages of the registry. All patients with SAEs and AEs must be monitored to determine the outcome. The trial must be reported to the principal investigator within 24 hours after the occurrence of SAEs and AEs. The initial report must be as complete as possible, including detailed information on the current SAEs and AEs in the trial (severe), as well as the assessment of the causal relationship between the events and the trial, and the occurrence of the events. The principal investigator will notify the trial monitor of serious SAEs upon request and is responsible for promptly reporting any serious SAEs or AEs to the ethics committee.

## 4 Discussion

This study aims to develop a mobile health intervention program targeting parents’ caregivers, such as parents, to improve the health management outcomes of patients with PKU. If the effectiveness of this program is validated, several key factors may contribute to its success. First, the program is based on the internationally recognized health intervention theories. In this research, the BCW theory and the family health theory framework are integrated. According to the six domains of family health, the BCW theory is extended to the family level. Following the structured steps of the BCW theory, the main obstacles affecting the family health behavior of PKU patients are analyzed, and appropriate intervention options are selected. Second, the program has formulated detailed, convenient, and diverse specific intervention measures. Guided by the nine intervention functions and seven policy categories of the BCW theory, the study designs a range of intervention strategies. And combined with the WeChat platform, various educational materials such as paper, pictures, and videos are designed to improve the accessibility and convenience of the intervention. Finally, the program carefully considers multiple factors that influence the outcomes. Based on existing research, this study not only examines the impact of the intervention program on metabolic, functional, and intellectual development indicators in PKU patients but also considers its effects on family members’ mental health, parenting stress, and other family-related factors. This comprehensive approach aims to design effective and minimally burdensome intervention measures, thereby facilitating the broader implementation of the program.

If this research plan proves effective, it can be extended to the health management of PKU patients in other regions of China. According to the data from the Chinese newborn screening information system from 2013 to 2017 (52), Gansu, Qinghai, Ningxia and Shanxi are among the provinces with the highest PKU incidence rates. Building on the development of PKU patient families in Inner Mongolia, a northern province, this study provides a set of referable intervention strategies for the health management of PKU patients in these provinces. Existing studies have established network-based programs for the health management of PKU patient families (35). These mobile health intervention platforms can be integrated with the theoretical model developed in this study to provide more personalized, convenient, efficient and cost-effective intervention plans for PKU patient families.

This study also has several limitations. First, due to the nature of the intervention, blinding of the interventionists is not feasible. To mitigate this limitation, detailed training and standardized intervention manuals will be provided to minimize subjective bias among intervention personnel, thereby ensuring consistency in the implementation of intervention measures. Second, some outcome measures rely on self-reported date from participants, which may introduce bias and affect the reliability of the results.
